# APSIC guide for prevention of Central Line Associated Bloodstream Infections (CLABSI)

**DOI:** 10.1186/s13756-016-0116-5

**Published:** 2016-05-04

**Authors:** Moi Lin Ling, Anucha Apisarnthanarak, Namita Jaggi, Glenys Harrington, Keita Morikane, Le Thi Anh Thu, Patricia Ching, Victoria Villanueva, Zhiyong Zong, Jae Sim Jeong, Chun-Ming Lee

**Affiliations:** Singapore General Hospital, Outram Road, Singapore, 169608 Singapore; Thammasat University Hospital, Bangkok, Thailand; Artemis Hospital, New Delhi, India; Infection Control Consultancy (ICC), Melbourne, Australia; Yamagata University Hospital, Yamagata, Japan; Cho Ray Hospital, Ho Chí Minh, Vietnam; Hong Kong Baptist Hospital, Kowloon, Hong Kong; Chong Hua Hospital, Cebu City, Philippines; West China Hospital of Sichuan University, Chengdu, China; Asan Medical Center, Seoul, Korea; MacKay Memorial Hospital, Taipei City, Taiwan

**Keywords:** Central line associated bloodstream infections, CLABSI, Insertion bundle, Maintenance bundle, Quality improvement

## Abstract

This document is an executive summary of the APSIC Guide for Prevention of Central Line Associated Bloodstream Infections (CLABSI). It describes key evidence-based care components of the Central Line Insertion and Maintenance Bundles and its implementation using the quality improvement methodology, namely the Plan-Do-Study-Act (PDSA) methodology involving multidisciplinary process and stakeholders. Monitoring of improvement over time with timely feedback to stakeholders is a key component to ensure the success of implementing best practices. A surveillance program is recommended to monitor outcomes and adherence to evidence-based central line insertion and maintenance practices (compliance rate) and identify quality improvement opportunities and strategically targeting interventions for the reduction of CLABSI.

## Background

Central line–associated bloodstream infections, or CLABSIs, are associated with increased morbidity, mortality, and health care costs [1]. It is now recognized that CLABSIs are largely preventable when evidence based guidelines are followed for the insertion and maintenance of Central Venous Catheters (CVC) [2]. The intent of this document is to highlight practical recommendations in a concise format designed to assist healthcare settings in the Asia Pacific region in implementing CLABSI prevention efforts. This document is a summary of the CLABSI prevention guidelines developed by the Asia Pacific Society of Infection Control (APSIC).

The term “central line” used in the guidelines is defined as an intravascular access device or catheter that terminates at or close to the heart or in one of the great vessels. The following are considered great vessels for the purpose of defining a central line; pulmonary artery, superior vena cava, inferior vena cava, brachiocephalic veins, internal jugular veins, subclavian veins, external iliac veins, common iliac veins or femoral veins. A hollow introducer is considered a central line if the tip is situated in a great vessel. The line may be used for infusion, or hemodynamic monitoring. Examples include a central venous catheter for infusion, pulmonary artery (PA) catheter, sheath/introducer for a PA catheter, dialysis or hemofiltration catheter in a great vessel and a peripherally inserted central catheter (PICC). A central line may be inserted centrally or peripherally (PICC) in a patient. Neither the location of the insertion site nor the type of device determines whether a line qualifies as a central line.

## Review

1. **Workgroup Composition**
  APSIC convened Infection Prevention and Control experts from the Asia Pacific region to develop the APSIC Guide for Prevention of Central Line Associated Bloodstream Infections (CLABSI). The members of this workgroup comprising key opinion leaders from the Asia Pacific region are the authors of this paper.
2. **Literature Review and Analysis**
  For the APSIC guideline, the workgroup reviewed previously published guidelines and recommendations relevant to each section and performed computerized literature searches using PubMed on keywords including CLABSI, CA-BSI, CR-BSI, Asia Pacific and guideline.
3. **Process**
  The workgroup met face to face on two occasions in addition to email correspondence to complete the development of the guideline. Discussion was also focused on how best to integrate the evidence in the Asia Pacific setting. Criteria for grading the strength of recommendations and quality of evidence are described in Table 1. Systematic review of existing guidelines was undertaken in addition to review of studies from the Asia Pacific region [3–14]. Expert consensus on selection of recommendations and strength of recommendations was obtained from the workgroup to develop the draft, which was then submitted to APSIC Executive Committee and national Infection Control societies in the Asia Pacific region. Comments obtained were then reviewed by the workgroup for necessary edits, following by final approval and endorsement by the APSIC Executive Committee and national societies from the Asia Pacific region.

**Table 1:** Categories for strength of each recommendation

Categories for strength of each recommendation	
Category	Definition
A	Good evidence to support a recommendation for use.
B	Moderate evidence to support a recommendation for use.
C	Insufficient evidence to support a recommendation for or against use
D	Moderate evidence to support a recommendation against use.
E	Good evidence to support a recommendation against use.
Categories for quality of evidence on which recommendations are made	
Grade	Definition
I	Evidence from at least one properly randomized, controlled trial.
II	Evidence from at least one well-designed clinical trial without randomization, from cohort or case-controlled analytic studies, preferably from more than one centre, from multiple time series, or from dramatic results in uncontrolled experiments.
III	Evidence from opinions of respected authorities on the basis of clinical experience, descriptive studies, or reports of expert committees.
4. **Recommendations for insertion**
  A. **The Central Line Insertion Bundle**
    1. Optimal site selection
    2. Hand hygiene
    3. Alcohol-based chlorhexidine skin preparation
    4. Maximum barrier precautions

## Optimal site selection [15–18]

The catheter insertion site affects the risk for catheter-related infection and phlebitis. The risk for catheter infection in part can be related to the risk for thrombophlebitis and the density of local skin flora. Femoral catheters are associated with a higher risk of infection and deep venous thrombosis, than internal jugular or subclavian catheters and should also be avoided, where possible. A subclavian site is preferred in adult patients and factors such as potential for mechanical complications and risk for subclavian vein stenosis, should be considered when determining the catheter insertion site.

1. *Select catheter type and insertion site on the basis of intended purpose and duration of use, risk of infectious and non-infectious complications (e.g., phlebitis and infiltration), and the skills and training of individuals inserting and caring for the central venous catheters. (IB)*
2. *Use a midline catheter or peripherally inserted central catheter (PICC), instead of a short peripheral catheter, when the duration of IV therapy will likely exceed six days. (IB)*
3. *Recommendations for central venous catheters*
  - *Weigh the risk and benefits of placing a central venous device at a recommended site to reduce infection against the risk of mechanical complications (e.g., pneumothorax, subclavian artery puncture, subclavian vein laceration, subclavian vein stenosis, hemothorax, thrombosis, air embolism, and catheter misplacement). (IA)*
  - *Avoid using the femoral vein for central venous access (I)*
  - *No recommendation can be made for a preferred site of insertion to minimize infection risk for a tunneled CVC. (UI Unresolved issue)*
  - *Place catheters used for short term hemodialysis and pheresis in a jugular or femoral vein, rather than a subclavian vein, to avoid venous stenosis. (IA)*
  - *Use ultrasound guidance when available to place central venous catheters to reduce the number of cannulation attempts and mechanical complications. (IB)*

## Hand hygiene [19, 20]

Hand hygiene before catheter insertion or maintenance, combined with proper aseptic technique during catheter manipulation and care, provides protection against infection.

1. *Hand hygiene should be performed before and after palpating catheter insertion sites as well as before and after inserting, replacing, accessing, repairing, or dressing an intravascular catheter site. Palpation of the insertion site should not be performed after the application of antiseptic, unless aseptic technique is maintained. (IB)*
2. *Maintain aseptic technique for the insertion and care of intravascular catheters. (IB)*
3. *Sterile gloves should be worn for the insertion of arterial, central, and midline catheters. (IA)*
4. *Use new sterile gloves before handling the new catheter when guidewire exchanges are performed. (II)*
5. *Wear either clean or sterile gloves when changing the dressing on intravascular catheters. (IC)*

## Alcohol-based chlorhexidine skin preparation [21, 22]

While alcohol-based chlorhexidine has become a standard antiseptic for skin preparation for the insertion of both central and peripheral venous catheters, alternatives may need to be used where there is a contraindication.

1. *Prepare and clean the skin site with an alcoholic chlorhexidine solution containing a concentration of 0.5 to 2 % chlorhexidine and 70 % alcohol before central venous catheter insertion and during dressing changes. If there is a contraindication to chlorhexidine* (e.g. hypersensitivity)*, tincture of iodine, an iodophor, or 70 % alcohol can be used as alternatives. (IA)*
2. *No recommendation can be made for the safety or efficacy of chlorhexidine in infants aged <2 months. (UI, unresolved issue).*
3. *Allow the skin antiseptic being used to dry completely before catheter insertion. (IB)*
4. *Disinfect catheter hubs, needleless connectors, taps and injection ports before accessing the catheter using an alcoholic chlorhexidine preparation or 70 % alcohol. (IIB)*

## Maximum barrier precautions [23]

These refer to the wearing a sterile gown, sterile gloves, mask and a cap along with the use of a full body sterile drape to cover the patient (similar to the sterile drapes used in the operating room) during the insertion of central venous catheters.

1. *Use maximal sterile barrier precautions during insertion of central venous catheters, (IB)*
2. *Use a sterile sleeve to protect pulmonary artery catheters during insertion. (IB)*

B. **Central line maintenance bundle components** [24–35]

CLABSI maintenance bundle components include:-

1. Daily review of line necessity and replacement
2. Hand hygiene
3. Disinfection of hubs
4. Strict aseptic technique for dressing changes
5. Standardize administration sets changes

## Daily review of line necessity and replacement

The central venous catheters should be reviewed daily for ongoing need. This is because the risk of CLABSIs increases with the duration of time the catheter is left in place, so daily evaluation of central lines is an important aspect of CLABSI prevention. Catheters that are no longer needed should be promptly removed.

To minimize the risk of infection:

1. *Designate only trained personnel who have demonstrated competency in the insertion and maintenance of central intravascular catheters. (IA)*
2. *Promptly remove any central venous catheter that is no longer required. (IA)*
3. *Ensure appropriate nursing staff levels in ICUs. Observational studies suggest that a higher proportion of “pool nurses” or an elevated patient–to-nurse ratio is associated with increased CRBSI in ICUs. (IB)*
4. *Promptly remove any intravascular catheter that is no longer required (IA)*
5. *When adherence to aseptic technique cannot be ensured (i.e. catheters inserted during a medical emergency), replace the catheter as soon as possible, i.e. within 48 hours. (IB)*
6. *Do not routinely replace CVCs, PICCs, hemodialysis catheters, or pulmonary artery catheters. (IB)*
7. *Do not remove CVCs or PICCs on the basis of fever alone. Use clinical assessment to determine if infection is evidenced elsewhere or if there is another non-infectious cause of the fever. (II)*
8. *Do not routinely change CVCs over guidewire exchanges for non-tunneled catheters. (IB)*
9. *Do not use guidewire exchanges to replace a non-tunneled catheter suspected of infection. (IB)*
10. *Use a guidewire exchange to replace a malfunctioning non-tunneled catheter if there is no evidence of infection is present. (IB)*

## Hand hygiene, glove use and aseptic technique

1. *Use new sterile gloves and aseptic technique before handling the new catheter when guidewire exchanges are performed. (II)*
2. *Hand hygiene should be performed before and after palpating catheter insertion sites as well as before and after inserting, replacing, accessing, repairing, or dressing an intravascular catheter. Palpation of the insertion site should not be performed after the application of antiseptic, unless aseptic technique is maintained. (IB)*
3. *Maintain aseptic technique for the insertion and care of intravascular catheters. (IB)*
4. *Wear either clean or sterile gloves when changing the dressing on intravascular catheters. (IC)*

## Disinfection of hubs and changing the access lumens/devices

The hubs on CVCs are a common source of bacterial colonization and serve as immediate portal of entry of microorganisms to the intraluminal surface of the catheter. These colonizers from the catheter hub and lumen can be dispersed into the bloodstream resulting in CLABSI. The disinfection of catheter hub surface is therefore, critical every time before they are accessed.

1. *Use a CVC with the minimum number of ports or lumens essential for the management of the patient. (IB)*
2. *No recommendation can be made regarding the use of a designated lumen for parenteral nutrition. (UI)*
3. *Change the needleless components at as the same time the administration set are changed or according to manufacturers’ recommendations for the purpose of reducing infection rates. There is no benefit to changing administration sets and hubs/connectors more frequently than every 72 h. (II)*
4. *Ensure that all components of the system are compatible to minimize leaks and breaks in the system. (II)*
5. *Minimize contamination risk by scrubbing the access port with an appropriate antiseptic (alcohol-based chlorhexidine, povidone iodine, an alcohol-based iodophor, or 70 % alcohol) and accessing the port only with sterile devices. (IA)*
6. *When needleless systems are used, a split septum valve may be preferred over some mechanical valves due to increased risk of infection with the mechanical valves. (II)*

## Proper dressing change technique

Transparent semipermeable dressings are preferred over gauze dressings as they allow continuous visual inspection of the catheter site. However, gauze dressings can be used if the patient is sweating or the site is bleeding or oozing following CVC insertion.

1. *Use either sterile gauze or sterile, transparent, semipermeable dressing to cover the catheter site. (IA)*
2. *If the patient is diaphoretic or if the site is bleeding or oozing, use a gauze dressing until this is resolved. (II)*
3. *Replace catheter site dressing if the dressing becomes damp, loosened, or visibly soiled. (IB)*
4. *Do not use topical antibiotic ointment or creams on insertion sites, except for dialysis catheters, because of their potential to promote fungal infections and antimicrobial resistance. (IB)*
5. *Do not submerge the catheter or catheter site in water. Showering should be permitted if precautions can be taken to reduce the likelihood of water reaching the catheter site (e.g., protect the catheter and administration connections and hubs with a waterproof cover during showering). This is because it increases the risk of organisms being introduced into the insertion site. (IB)*
6. *Replace gauze dressings as they become soiled. (II)*
7. *Replace transparent dressings used on CVC sites at least every 7 days, except in those pediatric patients in which the risk of dislodging the catheter may outweigh the benefit of changing the dressing. (IB)*
8. *Replace transparent dressings used on tunneled or implanted CVC sites no more than once per week (unless the dressing is soiled or loose), until the insertion site has healed. (II)*
9. *No recommendation can be made regarding the necessity for any dressing on well-healed exit sites of long-term cuffed and tunneled CVCs. (UI)*
10. * Ensure that catheter site care is compatible with the catheter material. (IB)*
11. * Use a chlorhexidine-impregnated sponge dressing for central venous catheters in patients older than 2 months of age if the CLABSI infection rate high and not decreasing despite adherence to maintenance bundle prevention measures, including education and training. (IB)*
12. * Encourage patients to report any changes in their catheter site or any new discomfort to staff. (II)*

## Standardize administration sets change

Administration sets are used for transfer of fluids, medicines and nutrition to patient’s body. Prolonged use of these sets increases the risk of infection. Therefore, routine change of the administration systems (primary and secondary sets and add-on devices) is recommended.

1. *In patients not receiving blood, blood products or fat emulsions, replace administration sets that are continuously used, including secondary sets and add-on devices, no more frequently than at 96-h intervals, but at least every 7 days. (IA)*
2. *No recommendation can be made regarding the frequency for replacing intermittently used administration sets. (UI)*
3. *No recommendation can be made regarding the frequency for replacing needles to access implantable ports. (UI)*
4. *Replace tubing used to administer blood, blood products, or fat emulsions (those combined with amino acids and glucose in a 3-in-1 admixture or infused separately) within 24 h of initiating the infusion. (IB)*
5. *Replace tubing used to administer propofol infusions every 6 or 12 hours, when the vial is changed, refer to the manufacturer’s recommendation. (IA)*

## Recommendations for implementation [36–40]

A key success factor to the implementation of the central line insertion and maintenance bundles is the adoption of the model of improvement approach involving multidisciplinary process stakeholders. The Plan-Do-Study-Act (PDSA) methodology to conduct small-scale tests of change in the ICU i.e. planning a test, trying it, observing the results, and acting on what is learned; is the scientific approach adopted in the implementation.

1. *Implementation of the use of the CLABSI insertion and maintenance bundles is best done using a quality improvement approach with a multidisciplinary team.*
2. *Build teams which include all staff involved in CVC insertion and maintenance including local champions.*
3. *Enhanced communication to share data and take action*
4. *Hospital leadership and policymakers are to continue providing support to build a culture of zero tolerance.*
5. *Lines of accountability need to be established to link everyone in a hospital - from the board to frontline staff - so that everyone has a shared understanding of the organizations goals, knows their role in meeting them, and receives feedback (e.g. dashboards) on how they are performing.*
6. *There should be an ongoing focus on skill development and competency assessment across the organization.*
7. *Education and training programs should be assessed for their content, relevance and impact on work performance.*
8. *Although adherence to evidence-based practices reduces inconsistencies in practice and can significantly improve the overall quality of care, healthcare organizations often find it difficult to implement best practices. Thus, identifying and removing barriers to adherence to these practices is essential to a successful implementation of best practices in the era of patient safety.*

C. **Additional measures to reduce CLABSI** [41–77]

The rationale for the use of chlorhexidine antiseptic bathing in place of soap and water bathing relates to the patient’s resident skin flora that can enter the bloodstream at the CVC insertion site or the extraluminal surface of the catheter. Reducing skin contaminants with chlorhexidine bathing can further reduce the risk of CLABSI.

Similarly, a chlorhexidine-impregnated dressing is now recommended by the Centers for Disease Control and Prevention (grade IB) when basic prevention measures are ineffective to decrease CLABSIs.

Additional measures to reduce infection include:

1. *Chlorhexidine bathing in addition to maximal barrier precautions and maintenance bundle prevention measures. (IIB)*
2. *If the CLABSI rate is not decreasing despite successful adherence to maintenance bundle prevention measures use a chlorhexidine-impregnated dressing at the catheter site in patients older than 2 months of age if there are no contraindications*
3. *Minocycline-rifampin or chlorhexidine-silver sulfadiazine impregnated catheters should be considered in adult patients whose catheter dwell time is expected to be >7 days and in units where the CLABSI infection rate is not meeting the set goal. (IA)*
4. *Patients using minocycline-rifampin or chlorhexidine-silver sulfadiazine-impregnated catheters should be monitored for side effects, such as anaphylaxis (IIIB).*
5. *Prophylactic antimicrobial or antiseptic lock solution should be considered for the following:*
  - *Patients with long-term hemodialysis catheters (IA)*
  - *Patients with limited venous access and a history of recurrent CLABSI (IIB)*
  - *Pediatric cancer patients with long-term catheters (IB)*
6. *Scrubbing the access port of connectors with an appropriate antiseptic and accessing the port only with sterile devices. (IA)*

## Surveillance

Surveillance for outcomes (CLABSI infection rates) is a primary outcome. Several centers have found it useful to monitor adherence to evidence-based central line insertion and maintenance practices (insertion bundle compliance rates) as a method for identifying quality improvement opportunities and strategically targeting interventions for the reduction of CLABSI.

1. The CLABSI rate are calculated per 1000 central line days
2. The Central line insertion bundle compliance rate is calculated as a percentage of central line insertions per month (%) [this is computed using data collected from checklist in Table 2]

**Table 2:** APSIC central line insertion checklist

Name of patient				Age		Sex		Unique ID		
Name of Insertor				Date		Time		Unit		
Is the Indication for insertion appropriate?									Yes	No
Type of Central Venous Catheter	Tunneled		Non-Tunneled		PICC line		Chemoport		Any other:	
Emergency Procedure	Yes	No								
*The Insertion Procedure*										
Was sublclavian or IJ vein the site for insertion - Y/N										
Has insertor and assistant performed hand hygiene procedures, either by washing hands with liquid soap and water or with alcohol-based hand rubs (ABHR)?									Yes	No
Was 70 % alcohol and >0.5 % CHG used in cleaning site of insertion?									Yes	No
Have both the operator and assistant practised maximal sterile barrier precautions (wearing a sterile gown, sterile gloves, and cap and using a full body drape for patient)?									Yes	No
Signature of person in-charge:

The Central line maintenance bundle compliance rate is calculated as a percentage of central line insertions per month (%) [This is computed using data collected from checklist in Table 3].

**Table 3:** APSIC central line maintenance checklist

Name of patient										Age			Sex					Unique ID			
Name of Treating physician/Surgeon																		Unit			
Type of Central Venous Catheter	Tunneled					Non-Tunneled			PICC line			Chemoport				Any other:					
Days	1			2			3			4			5			6			7		
Date																					
*The maintenance procedure*	Y	N	Comment:	Y	N	Comment:	Y	N	Comment:	Y	N	Comment:	Y	N	Comment:	Y	N	Comment:	Y	N	Comment:
			date for change in dressing, date of IV set change			date for change in dressing, date of IV set change			date for change in dressing, date of IV set change			date for change in dressing, date of IV set change			date for change in dressing, date of IV set change			date for change in dressing, date of IV set change			date for change in dressing, date of IV set change
Is review done for need for central line use?																					
Was hand hygiene practised before all line maintenance/access procedures?																					
Was alcohol used to disinfect hub before each access?																					
Was dressing changed using aseptic technique?																					
Were administration sets replaced every 4-7 days?																					
Signature of person in-charge:																					

Improvement takes place over time. Determining if improvement has really occurred and if it is a lasting effect requires observing rates of infection over time. Run charts can be used to monitor these changes. Run charts are graphs of data over time and are one of the single most important tools in performance improvement.

Feedback the data is best done in a timely manner to relevant clinical groups so that targeted CLABSI prevention and control measures can be introduced and reported on.

## Conclusion

There are few reports on the CLABSI rates in hospitals at Asia Pacific region. A recent systematic review revealed a pooled incidence density of 4 · 7 per 1000 catheter-days (95 % CI: 2 · 9- 6 · 5; I2 = 83 · 8; χ^2^*n* = 30 · 9, *p* < 0 · 001) from 6 published studies [78]. Most ICUs in developed countries now report CLABSI rates which are zero or close to zero. CLABSI is one of the most common and yet preventable healthcare associated infections. We recommend hospitals in the Asia Pacific region that have yet to achieve zero CLABSI rates continue surveillance of CLABSIs and implement Central Line Insertion and Maintenance Bundles using quality improvement approaches to improve practices as described in the APSIC Guide For Prevention Of Central Line Associated Bloodstream Infections (CLABSI).
